# Supplementary material for: Erythropoietin re-wires cognition-associated transcriptional networks
Source: Nat Commun. 2023 Aug 21;14:4777. doi: 10.1038/s41467-023-40332-8 (PMC10442354; doi:10.1038/s41467-023-40332-8)
Supplement: Supplementary file 1 — Supplementary Information [file 41467_2023_40332_MOESM1_ESM.pdf]

Supplementary Figures

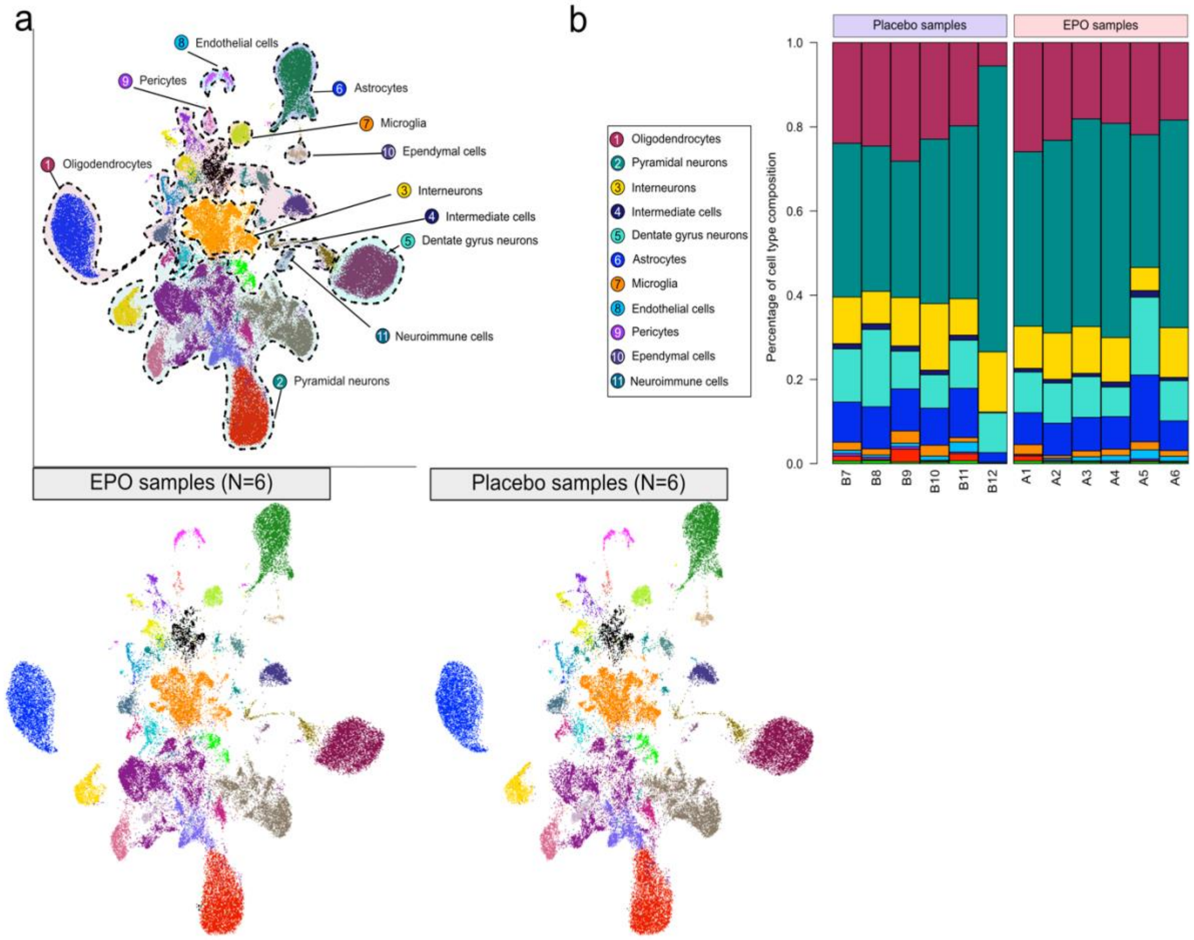

Supplementary Fig. 1: Computational efficacy of integrated snRNA-seq datasets.

a. UMAPs show the major neuronal and non-neuronal cell types from hippocampus by simultaneously integrating snRNA-seq datasets from EPO and PL samples using harmony.
b. The stacked barplot illustrates the composition of 10 major hippocampal and a neuroimmune cell type in each EPO and PL sample based on the annotations provided in Fig. 2b. Source data are provided on a repository[113] and as a Source Data file.

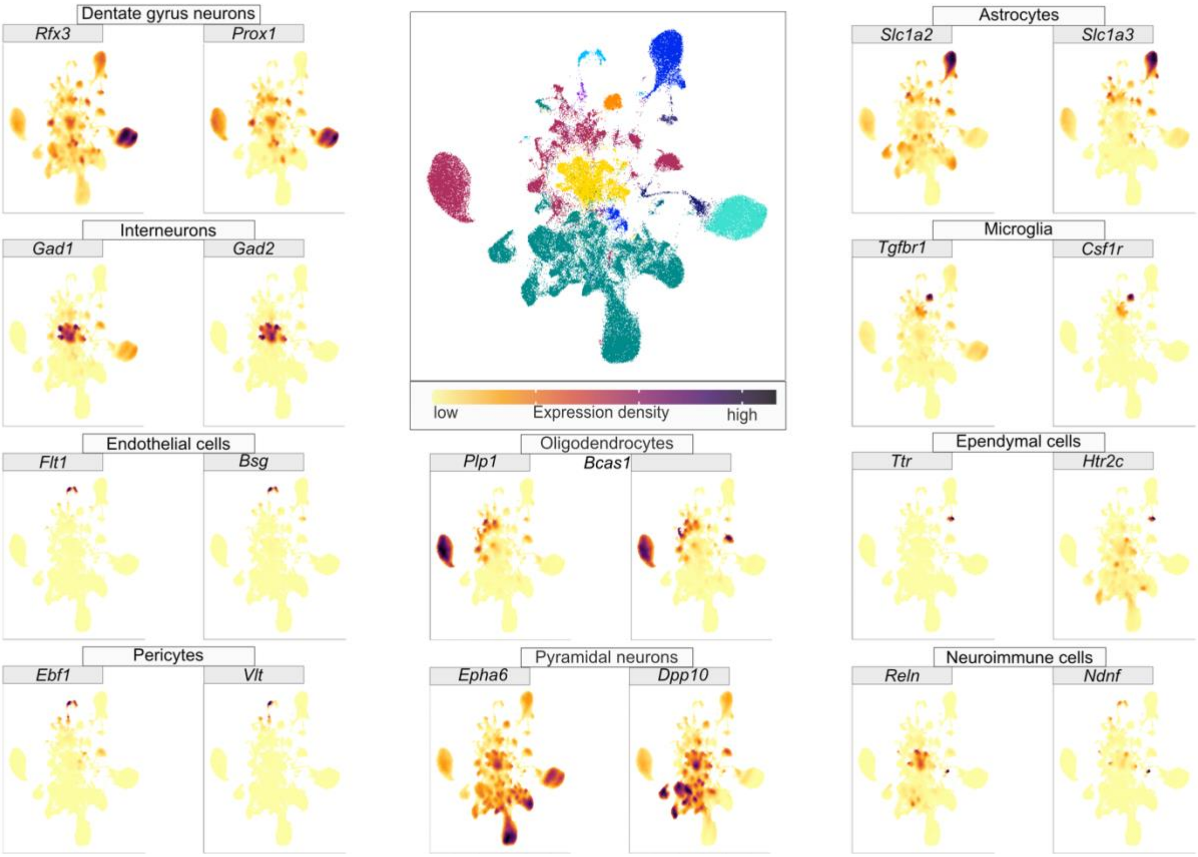

Supplementary Fig. 2: Annotation of hippocampal lineages using the bonafide markers.

Multiple feature plots based on UMAP displaying unsupervised identification of expression markers of neurons from Dentate gyrus (*Rfx3, Prox1*), interneurons (*Gad1, Gad2*), endothelial cells (*Flt1, Bsg*), pericytes (*Ebf1, Vlt*), oligodendrocytes (*Plp1, Bcas1*), pyramidal neurons (*Epha6, Dpp10*), astrocytes (*Slc1a2, Slc1a3*), microglia (*Tgfbr1, Csfr1*), ependymal cells (*Htr2c, Ttr*) and neuroimmune cells (*Reln, Ndnf*) (see Supplementary Data 3 for the full list of the markers). Dots in gold/maroon denote lower/higher expression in each single nuclei, respectively. Source data are provided on a repository[113] and as a Source Data file.

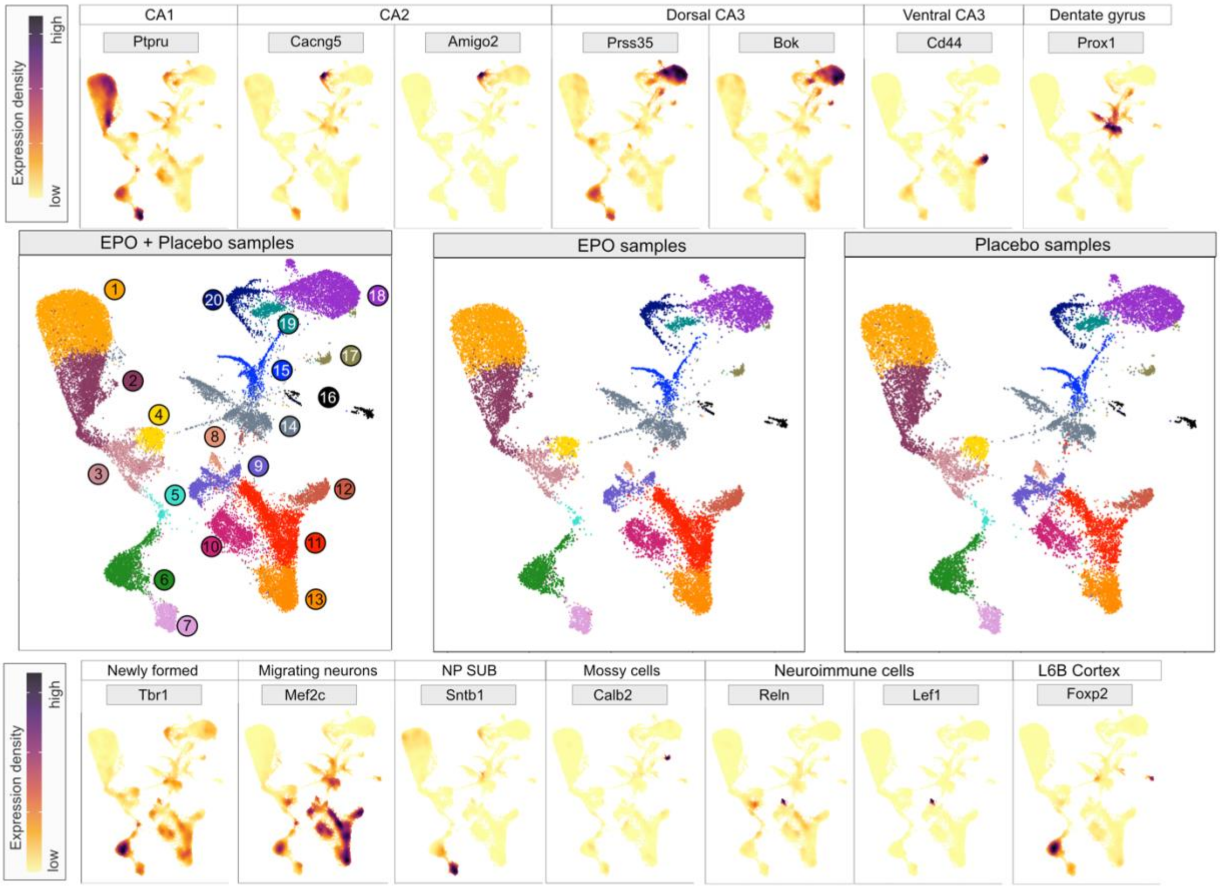

Supplementary Fig. 3: Annotation of pyramidal lineages using the bonafide markers.

Multiple feature plots based on UMAP of pyramidal clusters in EPO and PL displaying unsupervised identification of expression markers of CA1 (*Ptpru*), CA2 (*Cacng5, Amigo2*), CA3-dorsal (*Prss35, Bok*), CA3-ventral (*Cd44*), neurons from Dentate gyrus (*Prox1*), newly born/formed (*Tbr1*), migrating neurons (*Mef2c*), NP SUB neurons (*Sntb1*), mossy cells (*Calb2*), L6b cortex (*Foxp2*), and neuroimmune (*Reln, Lef1*) (see Supplementary Data 4 for the full list of the markers). Dots in gold/maroon denote lower/higher expression in each single nuclei, respectively. Source data are provided on a repository[113] and as a Source Data file.

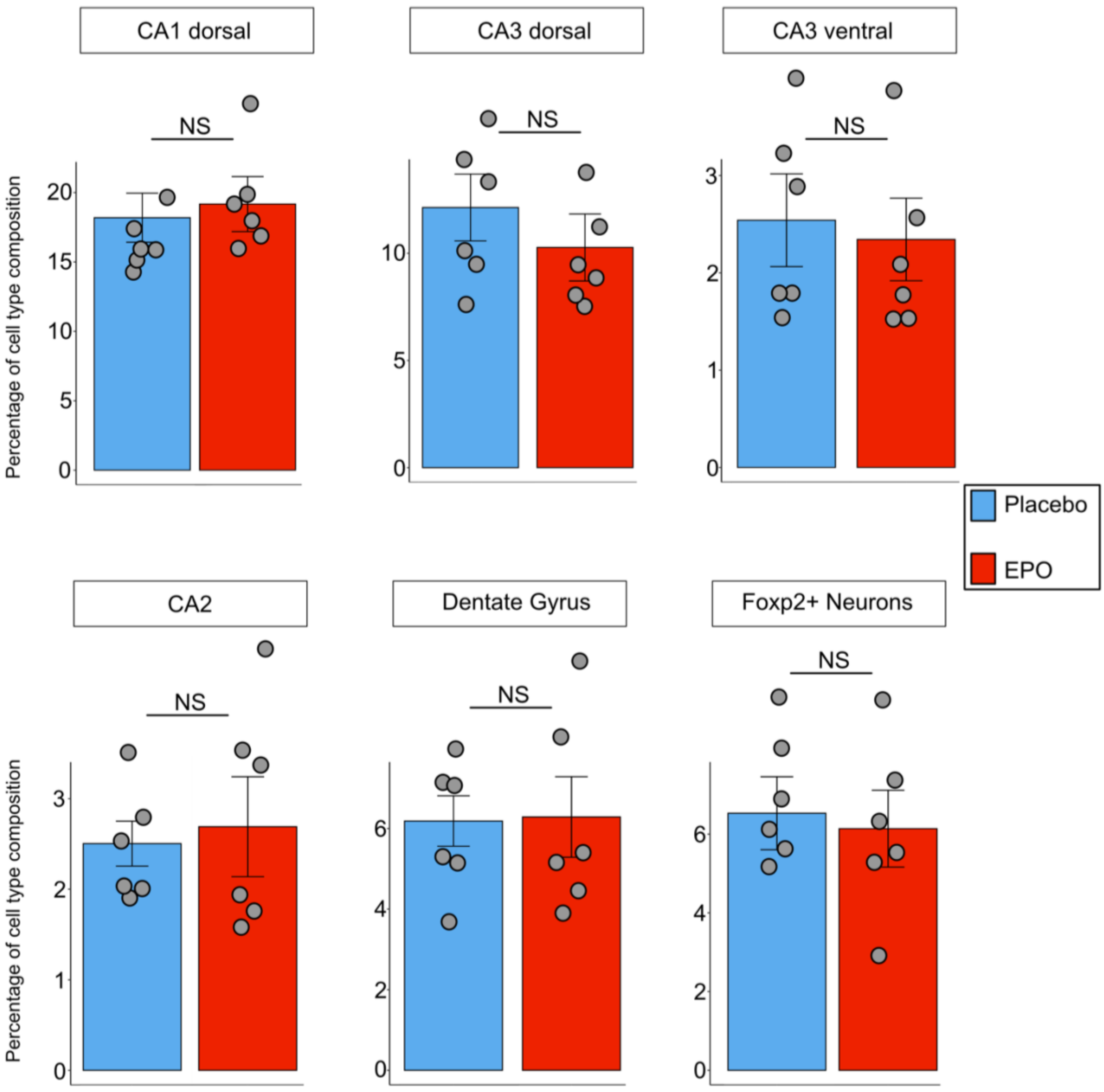

Supplementary Fig. 4: Relative abundances of pyramidal lineages between EPO and PL samples.

Barplots showing the relative abundance of pyramidal lineages in EPO and PL samples. The individual data points of six biological replicates corresponding to the lower, middle and upper limits of standard error bar shown on the plot. Grey circles on the bars represent the individual data points corresponding to each sample. Source data are provided on a repository[113] and as a Source Data file.

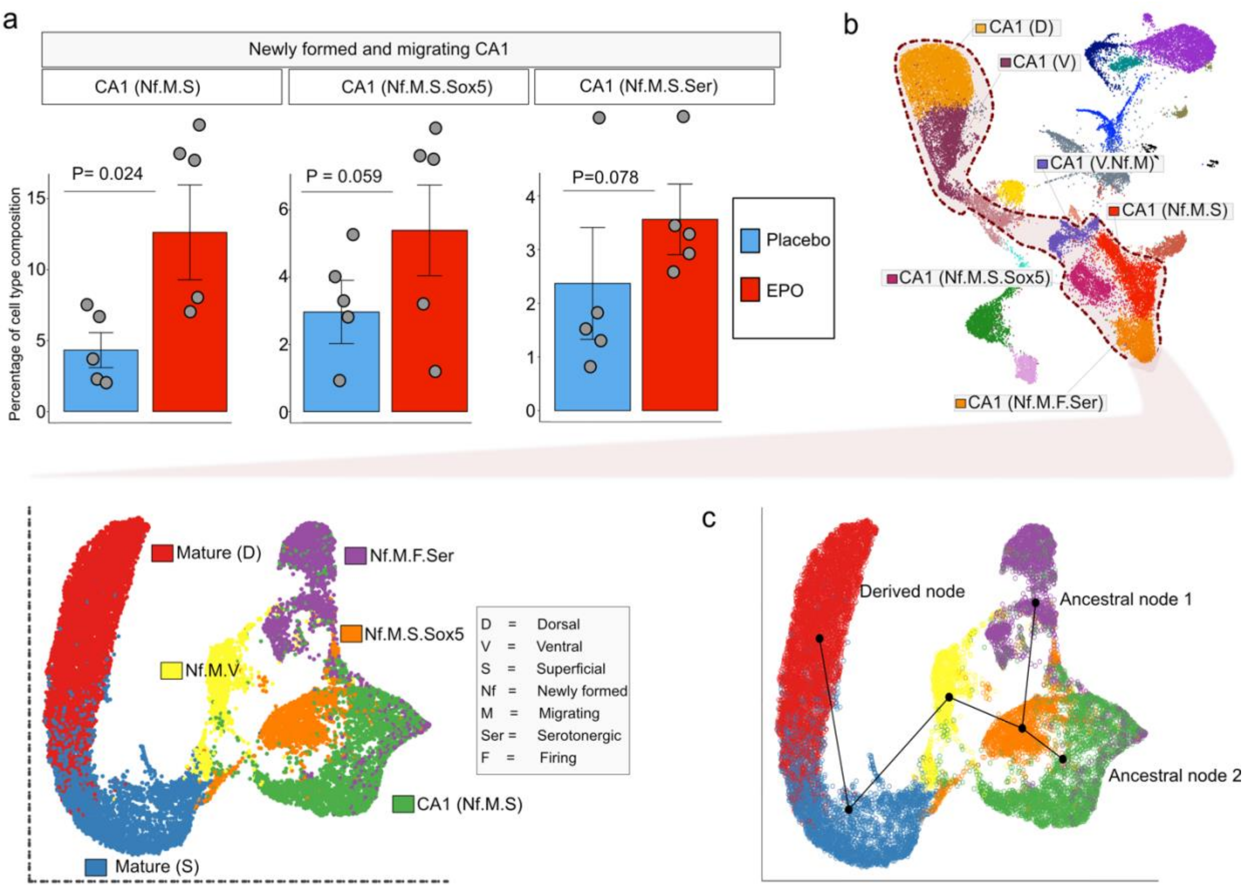

Supplementary Fig. 5: Overpopulation of newly-formed pyramidal neurons upon the EPO treatment

a. Barplots showing the relative differential abundance of 3 newly formed pyramidal lineages in EPO and PL samples. The individual data points of six biological replicates correspond to the lower, middle and upper limits of the standard error bar shown on the plot. P value is calculated using the Wilcoxon-rank-sum test. The grey circles on the bars represent the data points corresponding to each sample. Data are presented as mean ± SE fractions; n = 5. n indicates independent samples (two animals' hippocampus were pooled into one sample).
b. UMAP of selected lineages from pyramidal snRNA-seq data comprising mature CA1 neurons (dorsal and superficial), newly formed migrating - ventral (Nf.M.V), superficial Sox5+ and Sox5- (Nf.M.S (Sox5) and Nf.M.S.), and serotonergic firing (Nf.M.F.Ser).
c. Slingshot trajectory analysis of the lineages is shown in Figures 4 and Supplementary Fig. 5b. This tree-based computational method predicts that newly formed neurons are the pre-lineage of mature CA1 neurons. Source data are provided on a repository[113] and as a Source Data file.

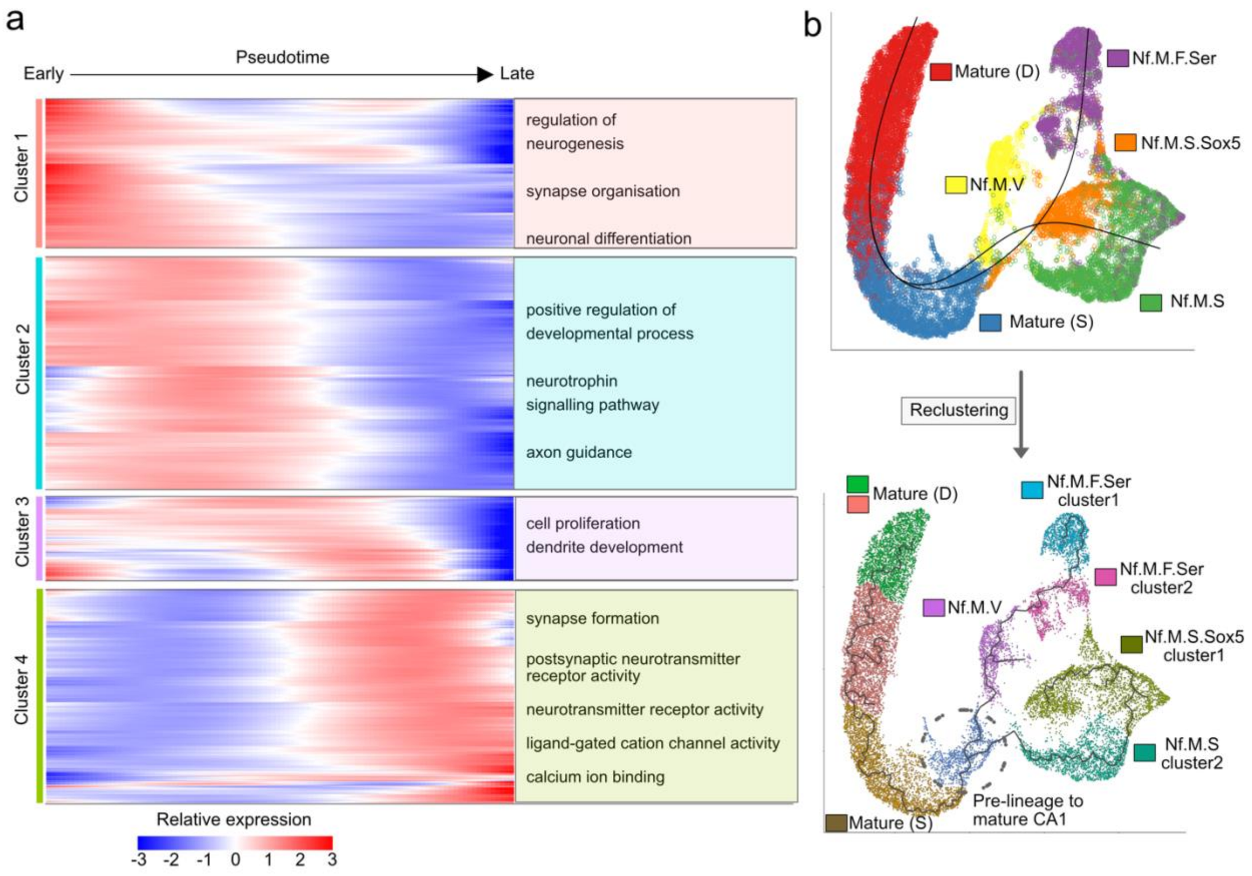

Supplementary Fig. 6: Pseudotime trajectory of pyramidal lineages pin the multiple progenitors as predecessors for mature CA1 neurons.

a. Heatmap showing the kinetics of highly and most variably expressed pyramidal genes changing gradually over the trajectory of newly formed to mature neurons from CA1 region. Genes (row) are clustered, and nuclei (column) are ordered according to the pseudotime progression.
b. Slingshot trajectory analysis of the lineages is shown in Figures 4 and Supplementary Fig. 5b-c (upper panel). In the lower panel, the same graph is reclustered in higher resolution space that further refines the subpopulations within the major cluster. The encircled cluster seems to be a common pre-lineage to the mature CA1 neurons that are newly formed with the expression of *Ctip2/Bcl11b* gene. Source data are provided on a repository[113] and s a Source Data file.

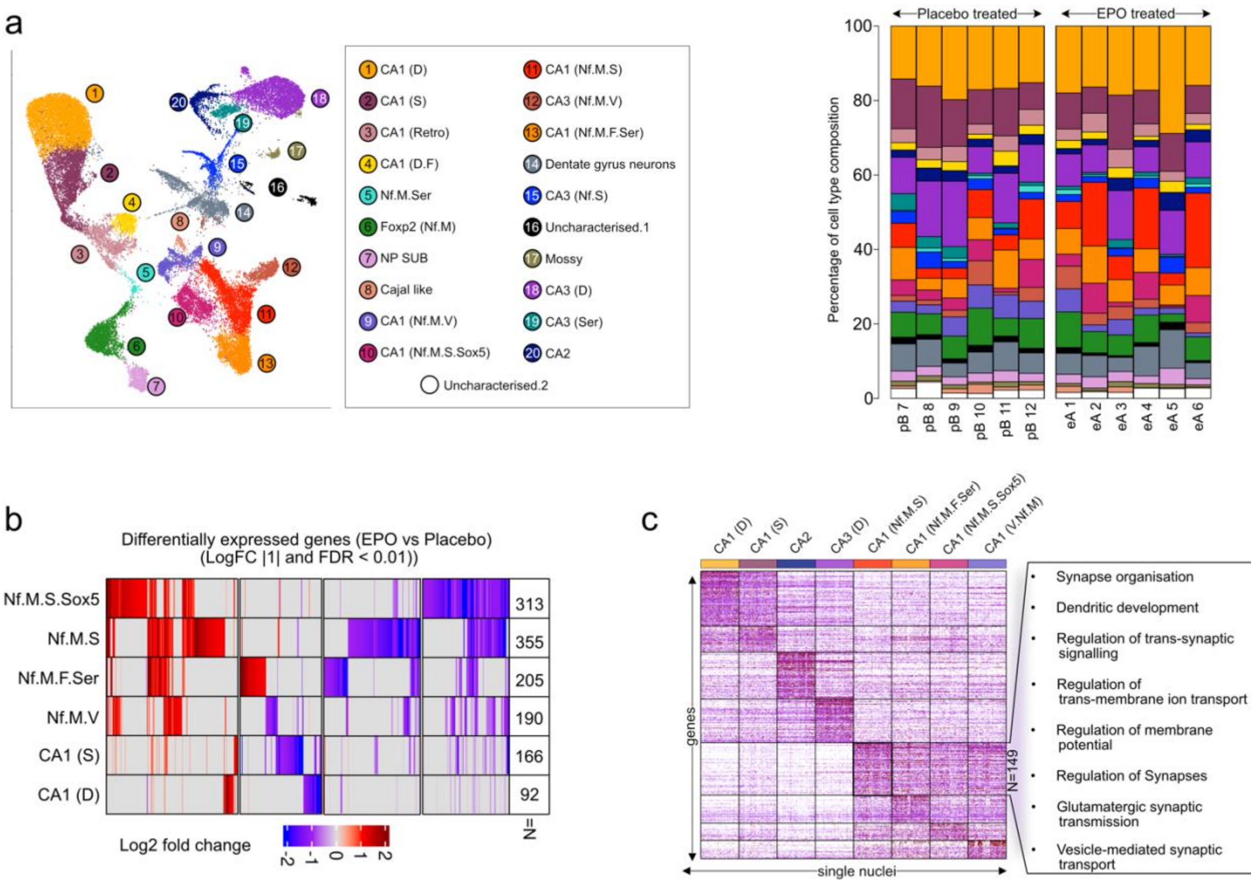

Supplementary Fig. 7: EPO treatment alters the transcriptional landscape of newly-formed pyramidal lineages.

a. UMAP of pyramidal lineages as shown in Fig. 3a with annotations enlisted in the box. This annotation is used only to analyze differential composition and gene expression in individual samples shown in Fig. 5b-c.
b. Heatmap representing differential expression of genes between EPO and PL samples in the individual lineages shown in Fig. 4. Only those genes that show a 2-fold change difference |2| and adjusted p-value <0.05 in any of the comparisons are shown here. Number of detected differentially expressed genes in each lineage is shown on the right side of the heatmap.
c. Heatmap showing scaled expression of distinctive marker gene sets defining CA1(D), CA1(S), CA2, CA3, and the 4 of CA1 newly formed migrating neuronal lineages. The color scheme is based on z-score distribution, from –2.5 (white) to 2.5 (purple). Boxed text beside the heatmap defines gene ontologies in which 149 genes, markers of newly formed migrating superficial lineage, are significantly enriched. Source data are provided on a repository[113] and as a Source Data file.

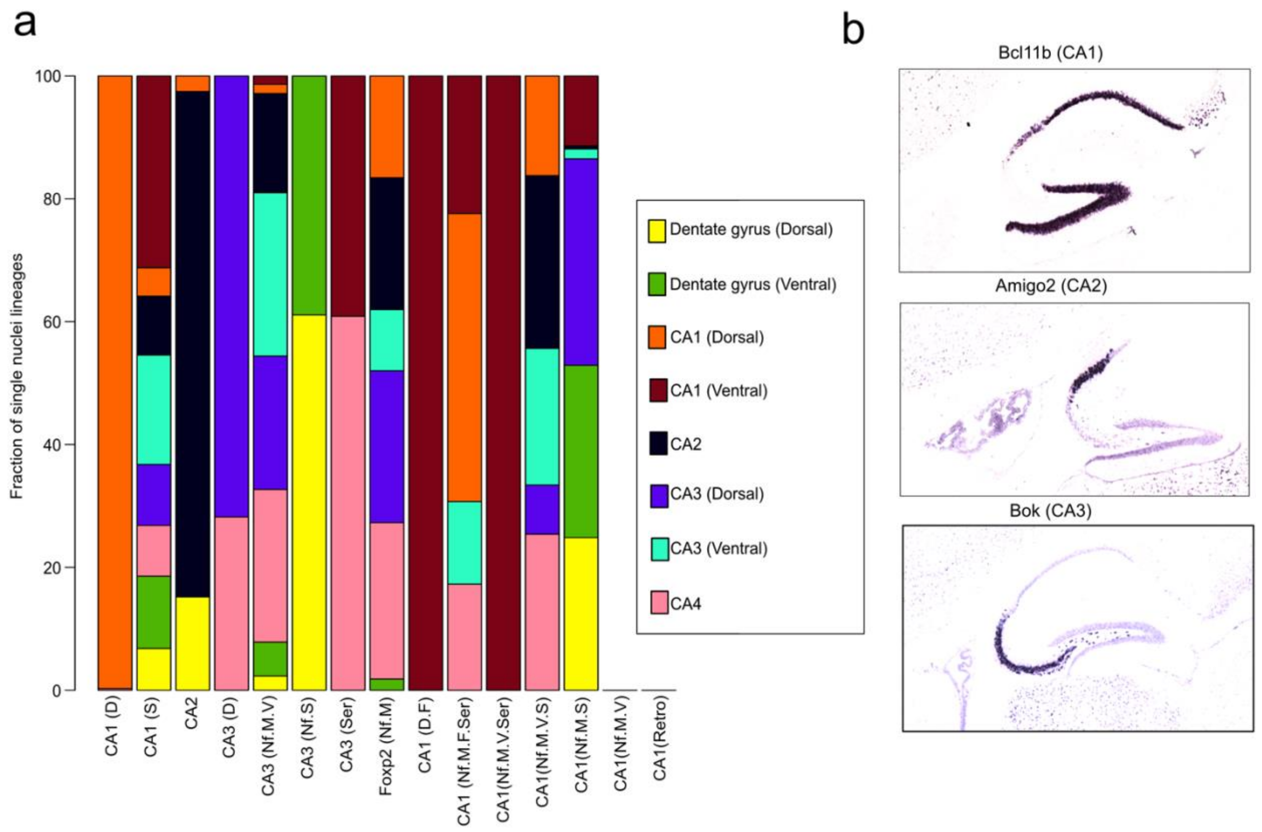

Supplementary Fig. 8: Evaluation of fidelity of lineages demonstrated in this study by comparing them with existing databases

a. Stacked barplot of obtained pyramidal lineages composition from deconvolution analysis of Hippocampal RNA-seq data. Data source: Hipposeq[34].
b. Screenshots of *in situ* hybridization results for the marker genes of CA1, CA2, and CA3 reported in this study. Data source: Allen brain gene expression atlas[90]. Source data are provided on a repository[113] and as a Source Data file.

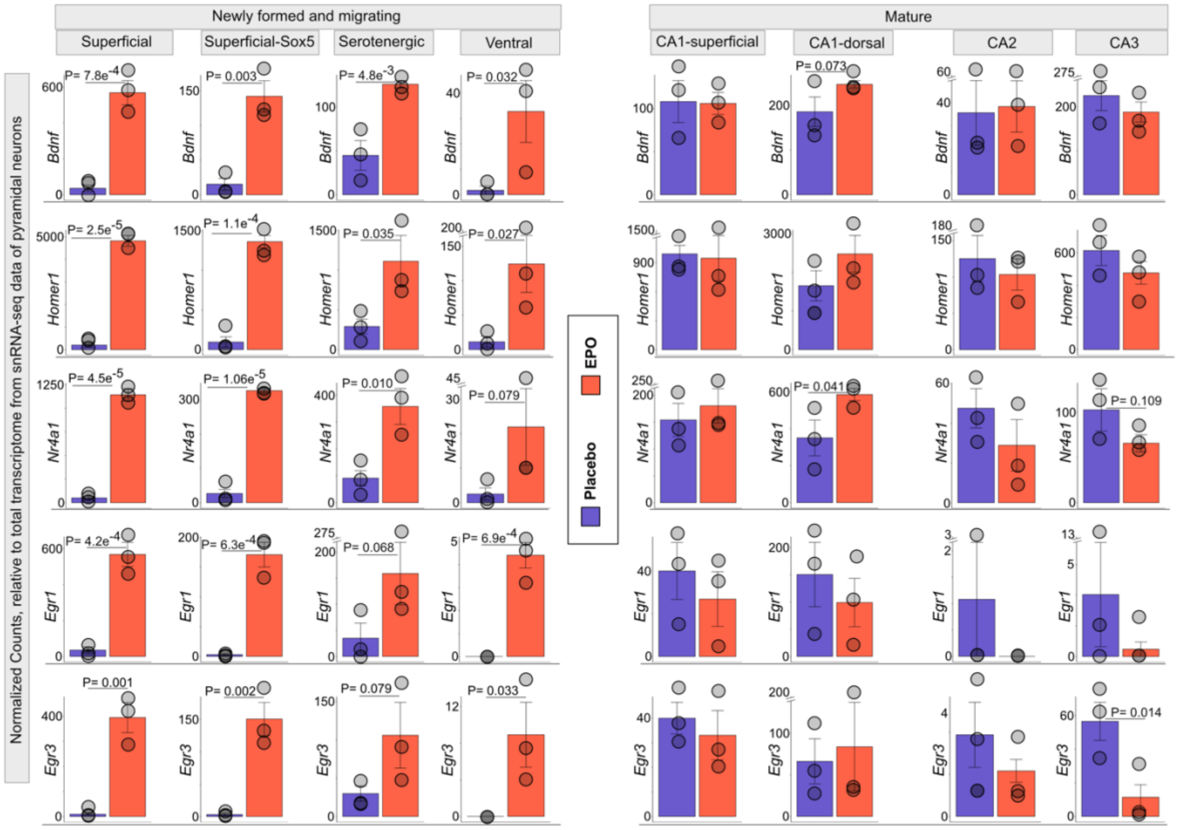

Supplementary Fig. 9 Expression pattern of individual neurotrophic or immediate early genes between EPO and PL samples, among the newly-formed and mature lineages.

Bar plots showing the selected neurotrophic and immediate early gene expression (normalised counts relative to transcriptome) levels in multiple pyramidal lineages. Data are presented as mean ± SE; n = 3. n indicates independent samples (two animals hippocampus were pooled into one sample). P value was determined by two-tailed unpaired Student’s t test. Source data are provided on a repository[113] and as a Source Data file.
