# Supplementary material for: Erythropoietin re-wires cognition-associated transcriptional networks
Source: Nat Commun. 2023 Aug 21;14:4777. doi: 10.1038/s41467-023-40332-8 (PMC10442354; doi:10.1038/s41467-023-40332-8)
Supplement: Supplementary file 3 — Description of Additional Supplementary Files [file 41467_2023_40332_MOESM3_ESM.pdf]

Description of Additional Supplementary Files

File Name: Supplementary Data 1
Description: Distinct gene expression units marking each of oligodendrocytes, intermediate cells, Dentate gyrus neurons, interneurons, pyramidal neurons, astrocytes, microglia, endothelial cells, pericytes, and ependymal cells from the total of 108,000 nuclei.

File Name: Supplementary Data 2
Description: Composition of each lineage shown in Fig. 2 from EPO and PL samples.

File Name: Supplementary Data 3
Description: Distinct gene expression units marking each cluster within the pyramidal lineage from ~36,000 nuclei.

File Name: Supplementary Data 4
Description: The relative abundance quantified on the level of fractions in each cluster of pyramidal lineage from EPO and PL.

File Name: Supplementary Data 5
Description: The relative abundance quantified on the fractions level in each of the analysed samples in every cluster of pyramidal lineage.

File Name: Supplementary Data 6
Description: 1043 genes (508 up, 535 down in EPO relative to PL) that are significantly altered (adjusted $p<0.05$). Next sheet within the same table contains all of the DEG found in this study.

File Name: Supplementary Data 7
Description: The list of all 36 regulons across the newly formed lineages is inferred from the SCENIC analysis.
