# Supplementary material for: Erythropoietin re-wires cognition-associated transcriptional networks
Source: Nat Commun. 2023 Aug 21;14:4777. doi: 10.1038/s41467-023-40332-8 (PMC10442354; doi:10.1038/s41467-023-40332-8)
Supplement: Supplementary file 11 — Reporting Summary [file 41467_2023_40332_MOESM11_ESM.pdf]

nature portfolio

Corresponding author(s): Hannelore Ehrenreich and Manvendra Singh

Last updated by author(s): Jun 23, 2023

Reporting Summary

Nature Portfolio wishes to improve the reproducibility of the work that we publish. This form provides structure for consistency and transparency in reporting. For further information on Nature Portfolio policies, see our Editorial Policies and the Editorial Policy Checklist.

Statistics

For all statistical analyses, confirm that the following items are present in the figure legend, table legend, main text, or Methods section.

n/a	Confirmed	
☐	☒	The exact sample size (*n*) for each experimental group/condition, given as a discrete number and unit of measurement
☐	☒	A statement on whether measurements were taken from distinct samples or whether the same sample was measured repeatedly
☐	☒	The statistical test(s) used AND whether they are one- or two-sided<br>*Only common tests should be described solely by name; describe more complex techniques in the Methods section.*
☐	☒	A description of all covariates tested
☐	☒	A description of any assumptions or corrections, such as tests of normality and adjustment for multiple comparisons
☐	☒	A full description of the statistical parameters including central tendency (e.g. means) or other basic estimates (e.g. regression coefficient) AND variation (e.g. standard deviation) or associated estimates of uncertainty (e.g. confidence intervals)
☐	☒	For null hypothesis testing, the test statistic (e.g. *F*, *t*, *r*) with confidence intervals, effect sizes, degrees of freedom and *P* value noted<br>*Give P values as exact values whenever suitable.*
☒	☐	For Bayesian analysis, information on the choice of priors and Markov chain Monte Carlo settings
☐	☒	For hierarchical and complex designs, identification of the appropriate level for tests and full reporting of outcomes
☐	☒	Estimates of effect sizes (e.g. Cohen's *d*, Pearson's *r*), indicating how they were calculated

Our web collection on statistics for biologists contains articles on many of the points above.

Software and code

Policy information about availability of computer code

Data collection

The details of the data generation are provided in the method sections of this manuscript. The data is available at the GSE220522. The computer code is provided as source file, is also on https://github.com/Manu-1512/Erythropoetin-says-Dracarys and on zenodo for citations

Singh, Manvendra. (2023). Erythropoietin rewires cognition-associated transcriptional networks. https://doi.org/10.5281/zenodo.8071471

Data analysis

The details of the data generation are provided in the method sections of this manuscript. Additionally, we provide all the computer codes on the GitHUB repository. The link can be found on
https://github.com/Manu-1512/Erythropoetin-says-Dracarys

10X Genomics CellRanger count v6.1.1 (To obtain gene/count matrix)
CellBender 0.2.1 (removal of background RNA)
Data analysis softwares are following
edgeR 3.42.4
slingshot 2.8.0
Nebulosa 1.10.0
harmony 0.1.0
R version 4.2.1

dplyr_1.0.10
monocle3_1.3.1

SingleCellExperiment_1.20.0
SummarizedExperiment_1.28.0
GenomicRanges_1.50.2
GenomeInfoDb_1.34.7
IRanges_2.32.0
Seurat_4.3.0
monocle_2.24.1
ggplot2_3.4.0
Biobase_2.58.0

igraph_1.3.5
magrittr_2.0.3
tensor_1.5
limma_3.54.0
tidyr_1.2.1
shiny_1.7.4

For manuscripts utilizing custom algorithms or software that are central to the research but not yet described in published literature, software must be made available to editors and reviewers. We strongly encourage code deposition in a community repository (e.g. GitHub). See the Nature Portfolio guidelines for submitting code & software for further information.

Data

Policy information about availability of data

All manuscripts must include a data availability statement. This statement should provide the following information, where applicable:

- Accession codes, unique identifiers, or web links for publicly available datasets
- A description of any restrictions on data availability
- For clinical datasets or third party data, please ensure that the statement adheres to our policy

Raw and processed data is deposited and the data is available at the GSE220522. The codes for the data analysis is available at https://github.com/Manu-1512/Erythropoetin-says-Dracarys, and on the zenodo repository

Singh, Manvendra. (2023). Erythropoietin rewires cognition-associated transcriptional networks. https://doi.org/10.5281/zenodo.8071471

Human research participants

Policy information about studies involving human research participants and Sex and Gender in Research.

Reporting on sex and gender	Not Applicable
Population characteristics	Not Applicable
Recruitment	Not Applicable
Ethics oversight	Not Applicable

Note that full information on the approval of the study protocol must also be provided in the manuscript.

Field-specific reporting

Please select the one below that is the best fit for your research. If you are not sure, read the appropriate sections before making your selection.

☒ Life sciences ☐ Behavioural & social sciences ☐ Ecological, evolutionary & environmental sciences

For a reference copy of the document with all sections, see nature.com/documents/nr-reporting-summary-flat.pdf

Life sciences study design

All studies must disclose on these points even when the disclosure is negative.

Sample size

This study includes 23 mice (N=11 EPO, N=12 PL) for the preparation of samples.
Here we were interested in resolving differences in means, i.e. a shift in the location parameter of distributions, by about one standard deviation (SD) or larger, i.e. Cohen's d >= 1, which corresponds to a "large" effect size according to Cohen's suggested interpretations.

With a recommended beta value (probability of a type II error) of <= 4 * alpha (probability of a type I error) (Cohen, 1969) and an alpha value of 0.05, we arrive at a statistical power (1-beta) >= 0.8. Required sample sizes for two-group and four-group comparisons were pre-calculated in R using the functions power.t.test() and pwr.anova.test(), respectively, from the R package 'pwr' version 1.3. For example, the required

sample sizes (per group) was n = 6 for an effect size of 1, i.e. a shift in means by one SD, and a power of 0.9. For a power of 0.8, the respective value was n = 5. For a smaller effect size of only 0.5, i.e. a shift in means by only 0.5 * SD, larger sample sizes of 17 (power = 0.8) and 22 (power = 0.9) are required. All n values represent biological replicates in this context, technical replicates do not apply.

References:

J Cohen (1969) Statistical Power Analysis for the Behavioral Sciences.
Erlbaum, Hillsdale, NJ, USA

R Core Team (2022) R: A language and environment for statistical
computing. R Foundation for Statistical Computing, Vienna, Austria.

Data exclusions	In one of the transcriptomics clustering analysis, we had found that one of the six sample was an outliar, so we removed it to obtain the robust set of DEGs.
Replication	Six total replication per condition. All n values represent biological replicates in this context, technical replicates do not apply.
Randomization	Not applicable because we used inbred strains.
Blinding	Not applicable because we used inbred strains.

Behavioural & social sciences study design

All studies must disclose on these points even when the disclosure is negative.

Study description	Not applicable
Research sample	Not applicable
Sampling strategy	Not applicable
Data collection	Not applicable
Timing	Not applicable
Data exclusions	Not applicable
Non-participation	Not applicable
Randomization	Not applicable

Ecological, evolutionary & environmental sciences study design

All studies must disclose on these points even when the disclosure is negative.

Study description	Not applicable
Research sample	Not applicable
Sampling strategy	Not applicable
Data collection	Not applicable
Timing and spatial scale	Not applicable
Data exclusions	Not applicable
Reproducibility	Not applicable
Randomization	Not applicable
Blinding	Not applicable

Did the study involve field work? ☐ Yes ☒ No

Reporting for specific materials, systems and methods

We require information from authors about some types of materials, experimental systems and methods used in many studies. Here, indicate whether each material, system or method listed is relevant to your study. If you are not sure if a list item applies to your research, read the appropriate section before selecting a response.

Materials & experimental systems

n/a	Involved in the study
☒	☐ Antibodies
☒	☐ Eukaryotic cell lines
☒	☐ Palaeontology and archaeology
☐	☒ Animals and other organisms
☒	☐ Clinical data
☒	☐ Dual use research of concern

Methods

n/a	Involved in the study
☒	☐ ChIP-seq
☒	☐ Flow cytometry
☒	☐ MRI-based neuroimaging

Antibodies

Antibodies used	Not applicable
Validation	Not applicable

Eukaryotic cell lines

Policy information about cell lines and Sex and Gender in Research

Cell line source(s)	Not applicable
Authentication	Not applicable
Mycoplasma contamination	Not applicable
Commonly misidentified lines (See ICLAC register)	Not applicable

Palaeontology and Archaeology

Specimen provenance	Not applicable
Specimen deposition	Not applicable
Dating methods	Not applicable

☐ Tick this box to confirm that the raw and calibrated dates are available in the paper or in Supplementary Information.

Ethics oversight	Not applicable

Note that full information on the approval of the study protocol must also be provided in the manuscript.

Animals and other research organisms

Policy information about studies involving animals; ARRIVE guidelines recommended for reporting animal research, and Sex and Gender in Research

Laboratory animals	C57BL6/N (WT) and NexCreERT2::R26R-tdT male mice received intraperitoneal injections (i.p.) of recombinant human (rh)EPO (5000 IU/kg body weight; NeoRecormon, Roche) or PL (solvent solution, 0.01 ml/g) every other day for 3 consecutive weeks starting on P28<br><br>Note: All males mice were sacrificed at P49 for snRNA-seq and NexCreERT2:TdTomato mice for Electrophysiology, All males were sacrificed between P49-P55

	All mice were housed in a temperature controlled environment (21 ± 2 °C) on a 12 h light–dark cycle with food and water available ad libitum.
Wild animals	No wild animals were used
Reporting on sex	Experiments performed on male mice
Field-collected samples	No field collected samples were used in this study
Ethics oversight	All experiments were approved by the local Animal Care and Use Committee (Niedersächsisches Landesamt für Verbraucherschutz und Lebensmittelsicherheit, LAVES) and conducted in accordance with the German Animal Protection Law.

Note that full information on the approval of the study protocol must also be provided in the manuscript.

Clinical data

Policy information about clinical studies

All manuscripts should comply with the ICMJE guidelines for publication of clinical research and a completed CONSORT checklist must be included with all submissions.

Clinical trial registration	Not applicable
Study protocol	Not applicable
Data collection	Not applicable
Outcomes	Not applicable

Dual use research of concern

Policy information about dual use research of concern

Hazards

Could the accidental, deliberate or reckless misuse of agents or technologies generated in the work, or the application of information presented in the manuscript, pose a threat to:

No	Yes	
☒	☐	Public health
☒	☐	National security
☒	☐	Crops and/or livestock
☒	☐	Ecosystems
☒	☐	Any other significant area

Experiments of concern

Does the work involve any of these experiments of concern:

No	Yes	
☒	☐	Demonstrate how to render a vaccine ineffective
☒	☐	Confer resistance to therapeutically useful antibiotics or antiviral agents
☒	☐	Enhance the virulence of a pathogen or render a nonpathogen virulent
☒	☐	Increase transmissibility of a pathogen
☒	☐	Alter the host range of a pathogen
☒	☐	Enable evasion of diagnostic/detection modalities
☒	☐	Enable the weaponization of a biological agent or toxin
☒	☐	Any other potentially harmful combination of experiments and agents

ChIP-seq

Data deposition

- [ ] Confirm that both raw and final processed data have been deposited in a public database such as GEO.
- [ ] Confirm that you have deposited or provided access to graph files (e.g. BED files) for the called peaks.

Data access links<br>*May remain private before publication.*	Not applicable
Files in database submission	Not applicable
Genome browser session<br>(e.g. UCSC)	Not applicable

Methodology

Replicates	Not applicable
Sequencing depth	Not applicable
Antibodies	Not applicable
Peak calling parameters	Not applicable
Data quality	Not applicable
Software	Not applicable

Flow Cytometry

Plots

Confirm that:

- [ ] The axis labels state the marker and fluorochrome used (e.g. CD4-FITC).
- [ ] The axis scales are clearly visible. Include numbers along axes only for bottom left plot of group (a 'group' is an analysis of identical markers).
- [ ] All plots are contour plots with outliers or pseudocolor plots.
- [ ] A numerical value for number of cells or percentage (with statistics) is provided.

Methodology

Sample preparation	Not applicable
Instrument	Not applicable
Software	Not applicable
Cell population abundance	Not applicable
Gating strategy	Not applicable

- [ ] Tick this box to confirm that a figure exemplifying the gating strategy is provided in the Supplementary Information.

Magnetic resonance imaging

Experimental design

Design type	Not applicable
Design specifications	Not applicable
Behavioral performance measures	Not applicable

Acquisition

Imaging type(s)	Not applicable
Field strength	Not applicable
Sequence & imaging parameters	Not applicable
Area of acquisition	Not applicable

Diffusion MRI ☐ Used ☒ Not used

Preprocessing

Preprocessing software	Not applicable
Normalization	Not applicable
Normalization template	Not applicable
Noise and artifact removal	Not applicable
Volume censoring	Not applicable

Statistical modeling & inference

Model type and settings	Not applicable
Effect(s) tested	Not applicable

Specify type of analysis: ☐ Whole brain ☐ ROI-based ☐ Both

Statistic type for inference (See Eklund et al. 2016)	Not applicable
Correction	Not applicable

Models & analysis

n/a	Involved in the study
☐	☒ Functional and/or effective connectivity
☐	☒ Graph analysis
☐	☒ Multivariate modeling or predictive analysis

Functional and/or effective connectivity	Spearmans correlation, ranked on the transcriptome-wide expression values
Graph analysis	All of the analysis is performed using the various pre-built packages in R. For details, see Method section of this paper.<br>Briefly, the first 30 PC dimensions were used in constructing the shared-nearest neighbor (SNN) graph and generating 2-dimension embeddings for data visualization using UMAP.<br>Pyramidal Neurons were then sorted for trajectory and pseudotime analysis using Monocle2 and Slingshot.<br>Regulons were found using the SCENIC package. Each contained at least 15 genes based on AUCell scores.
Multivariate modeling and predictive analysis	There are various multivariate analysis is presented here. All of the analysis is detailed in our method section and on GitHub link. For details, see Method section of this paper
